# GalaxyRefineComplex: Refinement of protein-protein complex model structures driven by interface repacking

**DOI:** 10.1038/srep32153

**Published:** 2016-08-18

**Authors:** Lim Heo, Hasup Lee, Chaok Seok

**Affiliations:** 1 Department of Chemistry, Seoul National University, Seoul 08826, Republic of Korea

## Abstract

Protein-protein docking methods have been widely used to gain an atomic-level understanding of protein interactions. However, docking methods that employ low-resolution energy functions are popular because of computational efficiency. Low-resolution docking tends to generate protein complex structures that are not fully optimized. GalaxyRefineComplex takes such low-resolution docking structures and refines them to improve model accuracy in terms of both interface contact and inter-protein orientation. This refinement method allows flexibility at the protein interface and in the overall docking structure to capture conformational changes that occur upon binding. Symmetric refinement is also provided for symmetric homo-complexes. This method was validated by refining models produced by available docking programs, including ZDOCK and M-ZDOCK, and was successfully applied to CAPRI targets in a blind fashion. An example of using the refinement method with an existing docking method for ligand binding mode prediction of a drug target is also presented. A web server that implements the method is freely available at http://galaxy.seoklab.org/refinecomplex.

Protein-protein interactions play critical roles in various biological processes, including enzyme catalysis^1^, cellular signal transduction^2^, and macromolecular assembly^3^. Three-dimensional protein-protein complex structures can provide atomic-level insights that can improve our understanding of protein-protein interactions and facilitate the engineering of proteins or small molecules with desired binding properties^4^. However, the number of co-crystalized protein-protein complex structures is still limited due to difficulties posed by experimental approaches. For example, experimentally determined three-dimensional protein-protein complex structures in the human proteome cover less than 10% of known interactions^5^,^6^. Therefore, accurate prediction of protein complex structures via *in silico* methods can be an effective alternative approach.

*In silico* prediction of protein-protein interactions was initially approached using rigid-body docking methods in which only the relative orientation between two proteins, represented by six translational and rotational degrees of freedom, is treated explicitly. The rigid-body docking problem can be solved efficiently using fast-Fourier transformation (FFT)^7^ or geometric hashing^8^ techniques. The internal flexibility of each protein structure is considered only implicitly, and low-resolution energy functions that allow some atomic overlaps are used, assuming that atomic overlaps or locally unfavorable interactions can be relaxed by local side-chain or backbone movement. Therefore, complex model structures generated by rigid-body docking may have some atomic clashes and may not have precise interface contacts^9^. Rigid-body docking methods are still effective for the generation of globally correct complex structures when conformational changes induced by binding are limited to local regions. Further refinement of the models generated by rigid-body docking using more computationally extensive flexible docking methods can therefore produce useful predictions for practical applications^9^,^10^,^11^,^12^,^13^,^14^,^15^,^16^,^17^.

Several methods for refinement of rigid-body docking model structures have been developed with some success by applying energy minimization or molecular dynamics simulations. RDOCK^9^ performs local energy minimizations on the protein-protein complex model structures generated by ZDOCK^7^, a rigid-body docking program. RDOCK was also extended by combining with ZRANK^18^. RosettaDock^12^ can refine protein-protein complex structures by the optimization technique of Monte Carlo with minimization. RosettaDock can optimize inter-protein orientation, side-chain conformation, and backbone conformation, such as loops^15^. Zhang *et al*.^16^ achieved effective conformational sampling with RosettaDock by applying an advanced sampling technique called well-tempered ensemble two dimensional Hamiltonian Replica Exchange Monte Carlo (WTE-H-REMC). HADDOCK^19^ performs energy optimization of interface side chains and backbone in torsion angle space first and then runs simulated annealing molecular dynamics (MD) simulations in Cartesian space with explicit waters. Król *et al*. applied nanosecond MD simulations^11^ to the refinement problem. The method iATTRACT^10^ performs energy minimization of the protein-protein complex structures generated by ATTRACT^20^ considering interface side-chain degrees of freedom for selected residues and rigid-body degrees of freedom. FiberDock^13^ and SymmRef^14^ employ normal modes to describe global backbone structure changes induced by binding for hetero-complexes and homo-complexes, respectively. Overall, the methods developed so far have been more successful in refining relatively high-accuracy models, and refinement of less accurate complex models or those involving inaccurate monomer structures, such as predicted structures, has yet to be achieved.

In this study, we introduce a new refinement method that improves less accurate protein-protein complex model structures compared to previous methods, for both hetero- and homo-complexes. This method, called GalaxyRefineComplex, was developed by extending the GalaxyRefine method for protein monomer structures^21^,^22^. GalaxyRefine successfully improved homology model structures in the blind protein structure prediction experiment Critical Assessment of techniques for protein Structure Prediction (CASP)^23^,^24^. GalaxyRefineComplex adapts the effective sampling method of GalaxyRefine by performing repetitive repacking of interface side chains followed by short MD relaxations. This sampling procedure mimics a protein-protein binding process in which side-chain interactions between two approaching proteins drive changes in the inter-protein orientation and intra-protein backbone conformation. The method was validated by refining models generated by ZDOCK^7^, M-ZDOCK^25^, and various methods used in the previous Critical Assessment of Prediction of Interactions (CAPRI)^26^,^27^ and CASP experiments. GalaxyRefineComplex was also successfully tested in a blind fashion in the CAPRI round 30^26^ (http://www.ebi.ac.uk/msd-srv/capri/round30/results/), which was held jointly with CASP11 (http://www.ebi.ac.uk/msd-srv/capri/round30/CAPRI_R30_v20141224.SW.pdf).

## Methods

### Overall procedure

**Figure 1:**
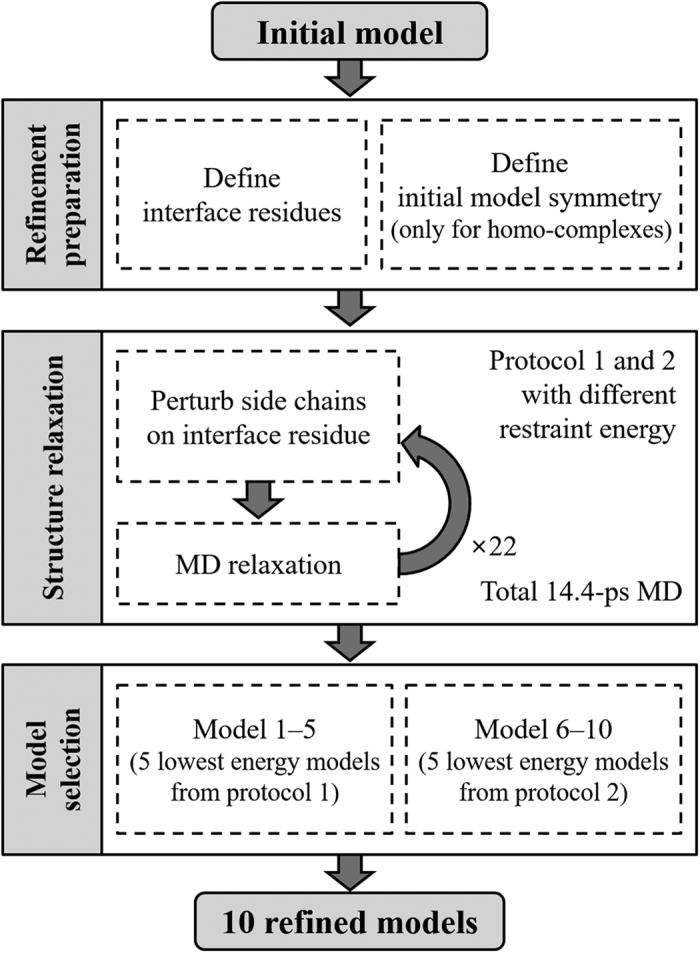
Flowchart of the GalaxyRefineComplex method.

A flowchart of the GalaxyRefineComplex method is provided in Fig. 1. This method is based on the GalaxyRefine method^21^,^22^ for structure refinement of single protein chains and was extended to protein-protein complexes. The refinement calculation starts with a local energy minimization and a 1.2-ps MD relaxation with a 4-fs time step, as in GalaxyRefine. According to our observation, energy minimization tends to generate very compact structures, and a short relaxation of 1.2 ps can create some physical space for atomic fluctuations, facilitating further conformational sampling. A major difference of GalaxyRefineComplex from GalaxyRefine is in the treatment of protein interfacial residues. Interfacial residues are defined here as those within 8 Å C_α_-C_α_ distance from any residue of the interaction partner. Relaxation of the input complex structure is driven by side-chain repacking of interfacial residues as follows. Interfacial residues are first repacked by three Monte Carlo (MC) steps of replacing the side-chain conformation of a cluster of up to five interfacial residues with a non-clashing rotamer conformation for three different clusters. Local side-chain conformation could be reasonably optimized by this short MC. A short MD relaxation of 0.6 ps is then performed with a 4-fs time step to allow overall conformational changes, including changes in the backbone and inter-protein orientation. During the Monte Carlo steps, the van der Waals radius is reduced to 70% to allow a small amount of clashes, which was proved effective in GalaxyRefine. The clashes can be relieved in subsequent relaxation steps. Side-chain repacking and relaxation is repeated 22 times (13.2-ps) because the relaxation was observed to converge after 10–15 ps. During the relaxation, the temperature is set to 300 K and is gradually decreased to 50 K for the last six steps (3.6 ps) as in simulated annealing to drive convergence to lower energy minimum without being trapped to nearby higher energy local minimum. Finally, local energy minimization is performed. For symmetric refinement of homo-complexes, symmetry transformation matrices for input chain structures are used to recover the symmetry after each MD step. The energy used for the MD relaxation and the criterion for model selection is explained in the following sections.

### GALAXY energy for complex refinement

The energy function used for MD relaxation (Eq. 1) is a linear combination of physics-based energy terms, knowledge-based energy terms, and restraint energy terms, with the relative weights of GalaxyRefine energy^21^,^22^, except for the restraint terms, as explained below.


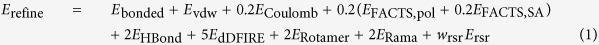


The physics-based terms include molecular mechanics bonded energy (*E*_banded_) and Lennard-Jones (*E*_vdw_) and Coulomb (*E*_Coulomb_) non-bonded interaction energy terms of CHARMM22^28^ with FACTS solvation free energy (*E*_FACTS,pol_ for the polar term and *E*_FACTS,SA_ for non-polar surface area term)^29^. The knowledge-based terms include hydrogen bond energy (*E*_HBond_)^30^, dipolar-DFIRE potential energy (*E*_dDFIRE_)^31^, and side-chain (*E*_Rotamer_) and backbone (*E*_Rama_) torsion angle energy^32^. The restraint energy terms include the following two components:


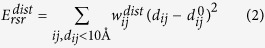



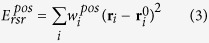


in which the reference distances 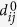 and the reference positions 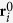 are taken from the input structure. The distance restraint of Eq. 2 is applied to all interface Cα-Cα and N-O atom pairs with distances *d*_*ij*_ < 10 Å with the same weight of 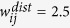 as used in GalaxyRefine for non-interface residues and with a much smaller weight of 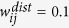 for interfacial residues to allow more structural changes on interface regions. Weak position restraint of Eq. 3 with a weight of 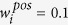 (compared to 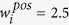 in GalaxyRefine) is applied to all Cα atoms to allow global changes in inter-protein orientation.

### Model generation and selection

Each of the two relaxation protocols, i.e., protocol 1, which applies only distance restraints (Eq. 2), and protocol 2, which applies both distance and position restraints (Eqs 2 and 3), is used to generate 16 structures by performing the relaxations described above 16 times. The five lowest-energy models out of the 16 models for each of protocols 1 and 2 are returned as 10 refined models. The five lowest-energy models from protocol 1 (and protocol 2) are ranked 1–5 (and 6–10) in the order of energy. This scheme for determining ranking among the 10 models, and in particular for selecting model 1, was determined by examination of the refinement results on the training set (constructed as explained in the next subsection) in terms of ligand RMSD (L-RMSD), interface RMSD (I-RMSD), fraction of predicted native contacts (F_nat_), and the MolProbity score (MolP), as summarized in Supplementary Table S1.

### Training and test sets

The refinement method was extensively tested on model structures of varying accuracies. First, models generated by ZDOCK^7^ for the ZDOCK benchmark 4.0 set complexes^33^ with unbound monomer structures were used to test the hetero-oligomer refinement method. Those generated by M-ZDOCK^25^ for the PISA benchmark set complexes^34^ with bound monomer structures were used to test the symmetric homo-oligomer refinement method. Only those complexes with less than 1,000 residues were considered for computational efficiency. For each complex, up to 1, 3, and 3 structures among models with high, medium, and acceptable accuracies, respectively (classified according to the CAPRI criterion)^27^, and up to three structures with the lowest L-RMSD among incorrect models with less than 15 Å L-RMSD were selected randomly. The number of model structures selected for each complex could be less than 10 because the number of models satisfying the above accuracy criteria could be less than the maximum number that could be selected.

Each of the ZDOCK and M-ZDOCK models was randomly divided into two subsets, and one subset was used as a training set to select model 1, as described in the previous subsection, and the other subset was used as a test set. The training set was composed of 643 ZDOCK models for 89 hetero-complex targets and 452 M-ZDOCK models for 46 homo-complex targets. The ZDOCK benchmark test set consisted of 677 models for 90 hetero-complex targets, and the PISA benchmark test set consisted of 445 models for 46 homo-complex targets.

Two additional test sets were constructed by collecting the model structures submitted during CAPRI blind prediction experiments. For each of the hetero-complex targets of CAPRI rounds 22, 24, 26, and 30 and the homo-complex targets of CAPRI round 30, up to 10 models showing varying accuracies were selected as described above. As a result, 34 models for five hetero-complex targets and 60 models for 13 homo-complex targets were selected. The models submitted by our own group (“Seok”) were excluded in these test sets because they were already refined by GalaxyRefineComplex. The blind prediction results of GalaxyRefineComplex on the CAPRI round 30 targets are presented separately.

### Comparison with existing methods

For performance comparison, available refinement docking methods, including RosettaDock, FiberDock, and SymmRef, were tested on the same sets. RosettaDock and FiberDock were used for refinement of hetero-complex models, and RosettaDock with the symmetry option and SymmRef were used for refinement of homo-complex models. For RosettaDock refinement^12^,^17^, the “docking_local_refine” protocol was applied to generate 1,000 structures with the extra χ1 (-ex1) and aromatic χ2 (-ex2aro) side-chain rotamer options for side-chain optimization. Among the 1,000 generated models, 10 models with the best energy values were selected. For homo-complex refinement by RosettaDock, a symmetry definition file generated by the “make_symmdef_file.pl” script for the initial complex structure was used to maintain symmetry. FiberDock and SymmRef were run with default parameters^13^,^14^. These methods generated a single refined model for each initial complex structure.

## Results and Discussion

### Performance comparison in terms of the CAPRI model accuracy criterion

The performance of GalaxyRefineComplex was compared with those of RosettaDock and FiberDock on the two hetero-complex sets (ZDOCK benchmark set and CAPRI set) and with those of RosettaDock run with a symmetry option and SymmRef, a symmetric version of FiberDock, on the two homo-complex sets (PISA benchmark set and CAPRI set). The accuracies of the initial models and refined models were classified using the CAPRI model quality criterion. The CAPRI criterion reflects the biological relevance of the model structures, and model qualities are classified as high (***), medium (**), acceptable (*), and incorrect considering L-RMSD and I-RMSD from the experimental structure and the F_nat_. The detailed criterion is as follows^27^: ‘high’ if L-RMSD or I-RMSD is lower than 1.0 Å with F_nat_ higher than 0.5, ‘medium’ if L-RMSD is lower than 5.0 Å or I-RMSD is lower than 2.0 Å with F_nat_ higher than 0.3, ‘acceptable’ if L-RMSD is lower than 10.0 Å or I-RMSD is lower than 4.0 Å with F_nat_ higher than 0.1, and incorrect for all other cases.

As shown in Table 1, GalaxyRefineComplex improved 114 of 263 incorrect models to acceptable or higher quality for the 677 ZDOCK models of the ZDOCK benchmark set by hetero-complex refinement, while RosettaDock improved 68 models for the same set when the best of 10 refined models was considered. When model 1’s were considered, only GalaxyRefineComplex succeeded in increasing the number of models with acceptable or higher quality, while RosettaDock and FiberDock failed. The numbers of high- and medium-quality models were also increased by GalaxyRefineComplex. RosettaDock and FiberDock were slightly better than GalaxyRefineComplex for refining models to high accuracy; both of the former methods improved five models to high accuracy, while GalaxyRefineComplex improved three.

When applied to the 34 hetero-complex models submitted during CAPRI experiments, GalaxyRefineComplex and RosettaDock improved eight and seven models, respectively, to acceptable or higher quality out of 15 incorrect models when the best of 10 refined models was considered (see Table 1). GalaxyRefineComplex also improved a larger number of incorrect models to acceptable or higher quality than RosettaDock and FiberDock when model 1’s were considered. Overall, GalaxyRefineComplex performed better than RosettaDock and FiberDock for improving incorrect or acceptable models to acceptable or medium accuracy and slightly worse for improving models to high accuracy when using the two hetero-complex test sets.

In the homo-complex refinement test on the 445 M-ZDOCK models of the PISA benchmark set, GalaxyRefineComplex showed a refinement performance that was similar to that of the hetero-complex refinement when both the best of 10 models and model 1’s were considered, as shown in Table 1. GalaxyRefineComplex improved a larger number of incorrect models to acceptable or higher quality than RosettaDock and SymmRef model 1’s were considered. However, RosettaDock and SymmRef performed much better than in the hetero-complex refinement test, improving more than 200 of 400 models to high accuracy, while GalaxyRefineComplex improved only 24 models. This seemingly different behavior on homo-complex refinement may be explained by the fact that the complex models of the PISA benchmark set were generated using the “bound” monomer structures because of the unavailability of unbound monomer structures. Therefore, this refinement set does not represent real case problems and can instead be considered an artificial set. Such problems may be relatively easy if shape complementarity is exploited intensively.

Refining of the 60 homo-complex models submitted during the CAPRI experiment was used to represent real case problems in which the initial models were generated by homology modeling. This test set, based on homology, is easier than the CAPRI hetero-complex set, with 58 of 60 initial models already having acceptable or higher quality. None of the tested methods could increase the number of acceptable or higher quality models in this case. However, GalaxyRefineComplex succeeded in refining five models to medium accuracy, while the other two methods failed.

### Refinement results in terms of ligand RMSD, interface RMSD, fraction of native contacts, and MolProbity score

**Figure 2:**
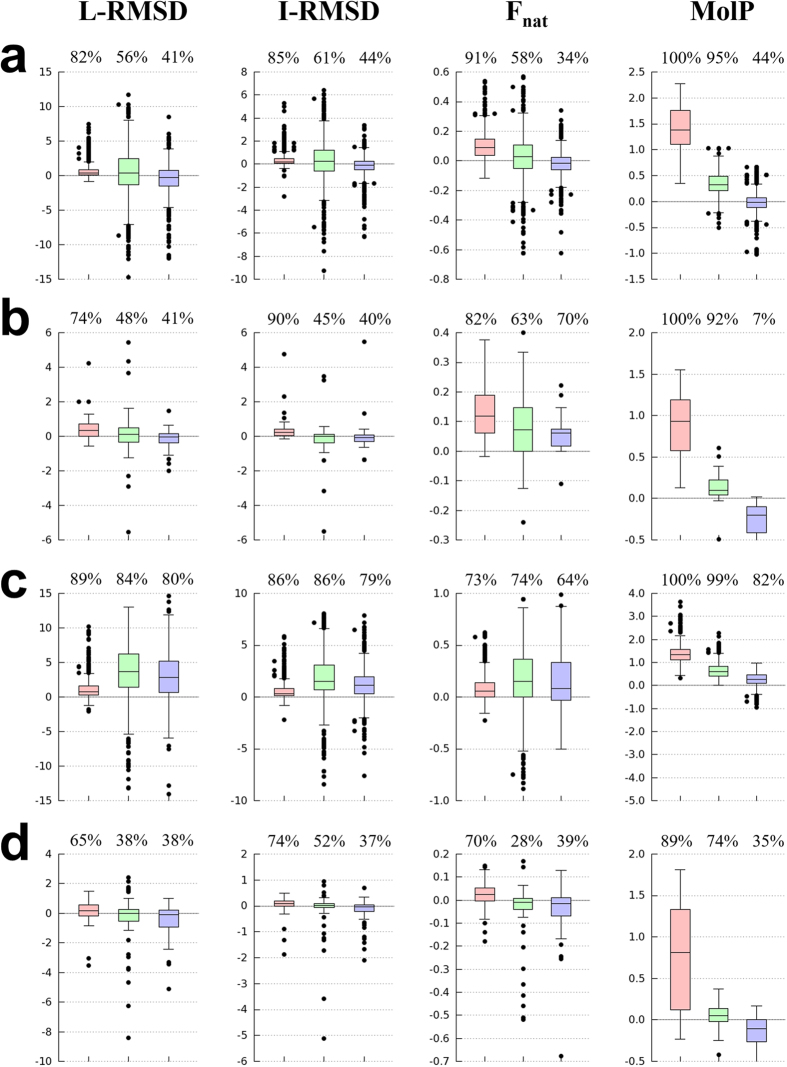
Quality comparison of the best out of 10 refined models generated by GalaxyRefineComplex (red) and RosettaDock (green) and the single refined models generated by FiberDock (blue in (**a**,**b**)) and SymmRef (blue in (**c**,**d**)) when the initial models were (**a**) ZDOCK models and (**b**) CAPRI models for hetero-complexes and (**c**) M-ZDOCK models and (**d**) CAPRI models for homo-complexes. The refinement results for different target complexes are depicted in boxplots, which present first and third quartiles as boxes and the median as the band inside the boxes. The minimum and maximum data within 1.5 interquartile range of the lower and upper quartile are represented as the bottom and top ends of whiskers, respectively, and data points outside of this range are shown with black dots as outliers. Note that only single models, not top ten models, were evaluated for FiberDock and SymmRef because these programs generate only single models.

Refinement results on the four test sets were analyzed in more detail using the three model quality measures of L-RMSD, I-RMSD, and F_nat_ used in CAPRI and MolP^35^, which measures physical incorrectness, such as the existence of steric clashes, Ramachandran outliers, and side-chain rotamer outliers. The results are summarized in Table 2. More detailed results for GalaxyRefineComplex are provided in Supplementary Tables S2–S4. The distributions of the values for the accuracy measures are also presented in Fig. 2 and Supplementary Figure S1.

When applied to the 677 ZDOCK models of the ZDOCK benchmark set, only GalaxyRefineComplex could improve model quality in all four measures on average. In contrast, RosettaDock only improved MolP, and FiberDock did not improve any models when model 1’s or the mean of final 10 models were considered. When the best of 10 refined models were considered, RosettaDock could improve models on average, but the extents of improvement for all measures were smaller than those of GalaxyRefineComplex. The statistical significance of the differences in L-RMSD and I-RMSD improvements by the two methods was not substantial, with *p*-values of 0.024 and 0.16, respectively, whereas that in F_nat_ and MolP was greater, with *p*-values of 6.5 × 10^−34^ and 1.1 × 10^−253^, respectively. GalaxyRefineComplex could improve initial models in 82%, 85%, 91%, and 100% of the cases in terms of L-RMSD, I-RMSD, F_nat_, and MolP, respectively, when the best of 10 models were considered.

In the refinement test on the 34 hetero-complex CAPRI models, GalaxyRefineComplex showed consistent improvement in all four quality measures on average, while RosettaDock and FiberDock did not, as shown in Table 2. The success of GalaxyRefineComplex for improving CAPRI models is more notable, considering that the CAPRI models may have already been refined by CAPRI predictors. Based on the data presented in the table, we concluded that complex models generated by the current docking methods could be easily improved at least in interfacial contacts and physical correctness. L-RMSD and I-RMSD may also be improved, but to a lesser extent.

For the 445 M-ZDOCK models of the PISA benchmark set that were generated with “bound” monomer structures, GalaxyRefineComplex could improve models in all measures, while RosettaDock could not improve in I-RMSD and SymmRef could not improve in MolP when the mean of the 10 models were considered (see Table 2). However, SymmRef showed the best performance in terms of the other three measures. The performance of RosettaDock was similar to that of GalaxyRefineComplex when model 1’s were considered, but significantly better when the best of 10 models were considered. This implied that RosettaDock scoring may need to be improved. The relatively poor performance of the GalaxyRefineComplex compared with that of the other two methods on model complex structures of “bound” monomer structures could be ascribed to the fact that only inter-protein orientations between receptor and ligand proteins needed to be adjusted in this case. GalaxyRefineComplex samples only interfacial residue conformations explicitly and allowed inter-protein orientations follow the conformational change of interfacial residues by short MD relaxation.

For the 60 homo-complex CAPRI models, all three methods tended to perform worse than for the other three test sets, as shown in Table 2. This set was the most difficult to refine because the monomer structures, based on homology, deviated more from the native structures than those of the other sets. Initial models of the ZDOCK benchmark set were constructed from unbound monomer structures resolved experimentally, those of hetero-complex CAPRI set from either unbound experimental structures or homology models, and those of the PISA set from bound structures. The overall quality of the initial complex models was better than in the other sets, as discussed in the previous subsection. Therefore, it may be difficult to improve the model quality without accounting for structural flexibilities of monomers at the interface. Despite this difficulty, GalaxyRefineComplex performed the best in all four measures among the compared methods, implying that the explicit sampling of interfacial residues and subsequent structural relaxation was effective.

**Figure 3:**
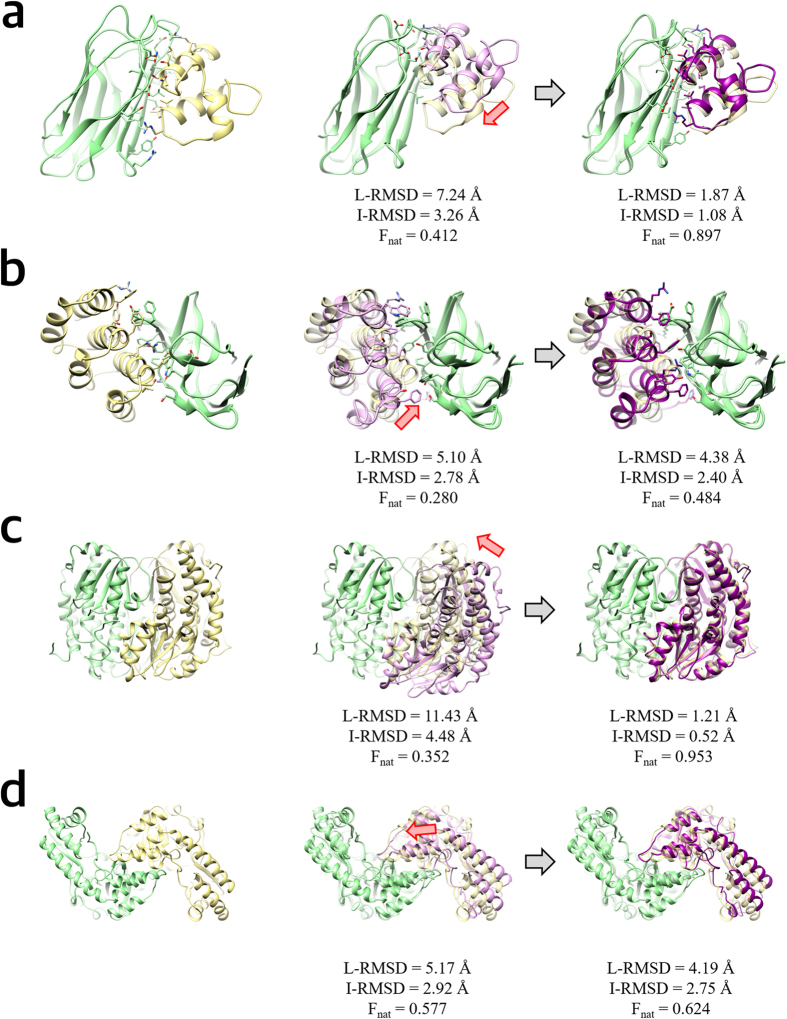
Successful refinement examples for hetero-complexes, i.e., (**a**) a model for TA12 of the ZDOCK benchmark 4.0 and (**b**) the model for CAPRI round 26 T54 submitted as P38_M07, and for homo-complexes, i.e., (**c**) a model for 1MOQ of the PISA benchmark set and (**d**) a model for CAPRI round 30 T87 submitted as TS417_2. In each panel, experimentally resolved structures are shown on the left, and model structures before and after refinement are shown in the middle and on the right, respectively. Receptor protein structures are depicted in green, ligand protein structures in experimental structures and in models before and after refinement are shown in yellow, pink, and violet, respectively. Red arrows indicate directions of changes in relative orientation made by refinement (from the pink to violet structures).

Successful refinement examples by GalaxyRefineComplex are illustrated in Fig. 3. A hetero-complex model generated by ZDOCK for the target TA12 of the ZDOCK benchmark 4.0^33^ was refined from acceptable to medium accuracy with improvement in L-RMSD from 7.24 to 1.87 Å, as shown in Fig. 3A. The initial model had 41% of native contacts with some voids at the interface and was refined to cover 90% of the native contacts. The hetero-complex model submitted in CAPRI round 26 for target 54 as P38_M07 was refined from acceptable to medium accuracy, as shown in Fig. 3B. Although improvements in RMSDs were small (<1 Å), native contacts increased by more than 20% through refinement. A homo-complex model generated by M-ZDOCK for one of the PISA benchmark set targets (PDB ID: 1MOQ) was refined dramatically from incorrect to medium accuracy, as shown in Fig. 3C. In another example, the model for target 87 of CAPRI round 30 submitted by group TS417 was refined from acceptable to medium accuracy, with improvement in L-RMSD from 5.17 to 4.19 Å (Fig. 3D).

### Blind prediction results of GalaxyRefineComplex in CAPRI round 30

GalaxyRefineComplex was used in CAPRI round 30 in a blind fashion under the group name “Seok”. Initial models generated using GalaxyGemini^36^, GalaxyLoop^37^, and other methods were subjected to refinement. Improvement by refinement is summarized in Table 3, and results for individual targets are provided in Supplementary Table S5. As in the test results reported in the previous subsection, L-RMSD and I-RMSD were improved by small magnitudes on average, whereas F_nat_ and MolP were improved more substantially.

### A practical example: accurate prediction of ligand binding mode of HIV-1 integrase by refinement

**Figure 4:**
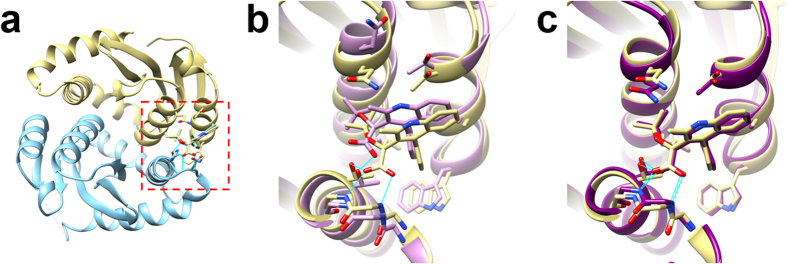
Ligand docking results for the dimer models of HIV-1 integrase generated with and without refinement by GalaxyRefineComplex. The full structure of the HIV-1 integrase dimer (PDB ID: 4NYF) and the ligand binding site at the dimer interface are shown in (**a**). Ligand docking results obtained with the dimer models before and after refinement are presented in (**b**,**c**), respectively. In (**b**,**c**), the crystal structure is colored in yellow. The predicted complex structures are colored in pink and blue, respectively. It can be seen that the predicted hydrogen bonds (cyan lines, generated using UCSF Chimera^43^) match perfectly with those of crystal structure in (**c**) but not in (**b**).

GalaxyRefineComplex was applied to the binding mode prediction of an inhibitor to HIV-1 integrase to illustrate the impact of complex refinement on practical applications such as drug discovery. HIV-1 integrase mediates integration of viral DNA into human DNA^38^ by forming a complex with a crucial co-factor, the human protein lens epithelium-derived growth factor (LEDGF)/p75. The co-factor binds at the dimer interface of HIV-1 integrase catalytic core domains, and inhibitors that bind at the same dimer interface can interfere with the co-factor binding. We first modeled the dimer structure by using M-ZDOCK with the monomer structure of HIV-1 integrase (PDB ID: 4DMN)^39^. GalaxyRefineComplex was then applied to generate a refined complex model. An inhibitor of HIV-1 integrase (PDB ID: 4NYF)^40^ was then docked by using the GalaxyDock^41^,^42^ protein-ligand docking program to the dimer model before and after refinement. Although the RMSD improvement of the model by refinement was rather mild (from 3.17 Å/1.40 Å/0.727 to 1.55 Å/1.09 Å/0.841 in L-RMSD/I-RMSD/f_nat_), the refinement lead to more dramatic improvement in the binding mode prediction (from 1.70 Å/36% to 0.60 Å/92% in ligand RMSD/percentage of protein-ligand contacts <5.0 Å). Moreover, the key interactions^38^,^40^ including the hydrogen bonds between protein backbone and ligand carboxyl group that were not predicted with the initial model could be predicted accurately with the refined model, as shown in Fig. 4.

### Origin of the effective refinement by GalaxyRefineComplex

GalaxyRefineComplex effectively refined hetero- and homo-complex model structures on the test sets, showing better results than the other refinement docking methods. Moreover, GalaxyRefineComplex consistently improved models constructed from monomer structures derived from either unbound experimental structures or homology models, unlike the compared methods. This requires additional computational cost, and the computer time as a function of protein size is provided in Supplementary Figure S2. GalaxyRefineComplex can improve docking models despite errors in the input monomer or interface structures due to the following two features. First, it uses a hybrid energy that consists of both physics-based and knowledge-based energy components. The physics-based energy terms contribute to improving the physical correctness of models, such as that measured by MolP. Knowledge-based energy, terms such as dipolar-DFIRE, makes the energy landscape smoother, allowing effective conformational sampling under an erroneous structural environment^37^. Knowledge-based energy also tends to better discriminate “native-like” conformations from decoys than physics-based energy^31^. Therefore, we believe that including those knowledge-based energy terms contributes to effective model selection. Second, intensive interface side-chain sampling before overall structure relaxation contributes to effective flexible refinement of docking models. According to our analysis, a refinement protocol without such intensive interface residue sampling performs much worse than the current protocol (see Supplementary Table S6 for detailed results). GalaxyRefineComplex mimics an actual process of conformational change induced by binding in which repacking of interfacial side chains drives further change to the conformation of the backbone.

## Conclusions

In this work, we presented a method for refining protein-protein complex structures generated by other docking programs. This method, called GalaxyRefineComplex, was compared with FiberDock, SymmRef, and RosettaDock on several sets of docking models with a range of initial model qualities. GalaxyRefineComplex showed consistent improvement, particularly for models of acceptable quality and for incorrect models. The method was able to improve model quality not only for unbound/bound structures but also for homology model structures, while the other methods were not. High-accuracy models could be improved mainly in contacts, whereas lower accuracy models could be refined both in contacts and relative inter-protein orientation. Repetitive side-chain repacking at the interface allows prediction of side-chain conformational change upon binding, contributing to improving contacts between interacting proteins. The knowledge-based energy terms of the GALAXY energy makes the method less sensitive to the accuracy of initial model qualities. The current method may be applied to various applications in which low- to medium-accuracy models are available but high-quality models are not. The program is freely available on the GalaxyWEB server at http://galaxy.seoklab.org/refinecomplex.

## Additional Information

**How to cite this article**: Heo, L. *et al*. GalaxyRefineComplex: Refinement of protein-protein complex model structures driven by interface repacking. *Sci. Rep.* **6**, 32153; doi: 10.1038/srep32153 (2016).

## Supplementary Material

Supplementary Information

## Figures and Tables

**Table 1:** Performance comparison of refinement methods in terms of the CAPRI accuracy criterion.

Hetero-complex refinement		
	Z**DOCK benchmark set** (677 models)	**CAPRI set** (34 models)
Initial models	414/18***/148**	19/1***/6**
GalaxyRefineComplex	528/25***/175** (475/21***/148**)	27/1***/8** (23/1***/7**)
RosettaDock	482/36***/171** (296/23***/86**)	26/2***/7** (19/2***/4**)
FiberDock^1^	–/–/– (390/23***/127**)	–/–/– (22/1***/4**)
Homo-complex refinement		
	**PISA benchmark set** (445 models)	**CAPRI set** (60 models)
Initial models	314/45***/134**	58/30**
GalaxyRefineComplex	389/90***/146** (364/69***/147**)	57/35** (57/35**)
RosettaDock	391/285***/52** (347/252***/39**)	52/29** (48/23**)
SymmRef^1^	–/–/– (361/283***/14**)	–/–/– (57/26**)

The numbers of targets for which the best of 10 refined models were of acceptable or higher accuracy/high accuracy (***)/medium accuracy (**) are presented, and those for model 1’s are shown in parentheses.

^1^Data for 10 models are not provided for FiberDock and SymmRef because they generate only single models.

**Table 2:** Performance comparison of different refinement methods in terms of mean improvement/percentage of improved cases in ligand RMSD (L-RMSD), interface RMSD (I-RMSD), fraction of native contact (F_nat_), and MolProbity score (MolP).

**Hetero-complex refinement**					
**Test set**	**Method**	−ΔL-RMSD (Å)	−ΔI-RMSD (Å)	ΔF_nat_ (%)	−ΔMolP
**ZDOCK benchmark set** (677 models)	GalaxyRefineComplex	0.68/82% (0.06/54%) <0.11/56%>	0.37/85% (0.15/59%) <0.12/64%>	10.3/91% (7.7/81%) <6.9/87%>	1.41/100% (1.33/100%) <1.32/100%>
	RosettaDock	0.40/56% (−3.73/29%) <−3.11/25%>	0.30/61% (−1.61/32%) <−1.33/26%>	2.2/58% (−8.4/30%) <−7.9/33%>	0.35/95% (0.33/95%) <0.32/95%>
	FiberDock^1^	–/– (−0.59/41%)	–/– (−0.19/44%)	–/– (−2.2/34%)	–/– (−0.02/44%)
**CAPRI set** (34 models)	GalaxyRefineComplex	0.48/74% (0.20/65%) <0.15/60%>	0.47/90% (0.02/68%) <0.12/60%>	11.0/82% (7.6/61%) <7.8/82%>	0.95/100% (0.85/98%) <0.84/100%>
	RosettaDock	−2.38/48% (−3.95/30%) <−3.80/30%>	−3.38/45% (−3.99/22%) <−4.29/20%>	7.2/63% (2.6/46%) <2.2/52%>	0.16/92% (0.13/81%) <0.13/84%>
	FiberDock^1^	–/– (−0.19/41%)	–/– (0.06/40%)	–/– (4.8/70%)	–/– (0.00/0%)
**Homo-complex refinement**					
**PISA benchmark set** (445 models)	GalaxyRefineComplex	1.30/89% (0.78/73%) <0.63/76%>	0.63/86% (0.37/72%) <0.32/73%>	8.5/73% (6.1/64%) <4.5/64%>	1.36/100% (1.25/100%) <1.25/100%>
	RosettaDock	3.49/84% (0.82/73%) <0.38/69%>	1.74/86% (0.17/73%) <−0.08/67%>	17.1/74% (6.6/61%) <1.9/55%>	0.66/99% (0.57/99%) <0.56/99%>
	SymmRef^1^	–/– (2.90/80%)	–/– (1.14/79%)	–/– (14.6/64%)	–/– (−2.55/0%)
**CAPRI set** (60 models)	GalaxyRefineComplex	0.08/65% (−0.05/60%) <−0.04/60%>	0.07/74% (0.02/71%) <0.01/65%>	2.6/70% (0.8/57%) <0.9/57%>	0.78/89% (0.73/83%) <0.72/83%>
	RosettaDock	−1.22/38% (−3.31/22%) <−3.97/20%>	−0.86/52% (−1.71/30%) <−2.19/25%>	−5.9/28% (−9.2/18%) <−11.6/15%>	0.06/74% (0.03/70%) <0.03/73%>
	SymmRef^1^	–/– (−0.43/38%)	–/– (−0.20/37%)	–/– (−4.3/39%)	–/– (0.00/0%)

Results for the best of 10 refined models are presented, and those for model 1’s and the mean of the 10 models are shown in parentheses and pointy brackets, respectively.

^1^Data for the 10 models and the mean of the 10 models are not provided for FiberDock and SymmRef because they generate only single models.

**Table 3:** Blind refinement results of GalaxyRefineComplex on the 13 initial models generated by GALAXY methods in CAPRI round 30 for the best of 10 submitted models.

CAPRI criterion			
Initial models	13/7**		
GalaxyRefineComplex	13/7**		
**Detailed measures** (mean improvement/percentage of improved cases)			
−ΔL-RMSD (Å)	−ΔI-RMSD (Å)	ΔF_nat_ (%)	−ΔMolP
0.12/62%	0.09/85%	0.075/85%	0.83/100%
