# Supplementary material for: GalaxyRefineComplex: Refinement of protein-protein complex model structures driven by interface repacking
Source: Sci Rep. 2016 Aug 18;6:32153. doi: 10.1038/srep32153 (PMC4989233; doi:10.1038/srep32153)
Supplement: Supplementary Information [file srep32153-s1.pdf]

Supporting Information

GalaxyRefineComplex: Refinement of protein-protein complex model structures driven by interface repacking

Lim Heo, Hasup Lee, and Chaok Seok

Department of Chemistry, Seoul National University, Seoul 08826, Republic of Korea

TABLES

Table S1. Refinement results obtained by protocol 1 and 2 are presented for the best of top 5 models (and the model 1 in parentheses). The better results among the two protocols are emphasized in bold.

**Training Set**		**No. models**	**Mean improvements by protocol 1/protocol 2**			
			ΔL-RMSD	ΔI-RMSD	ΔF_{nat}	ΔMolP
**ZDOCK models for hetero-complexes**	High	11	-0.12/**0.22** (-0.54/**0.13**)	-0.04/**0.01** (-0.12/**-0.02**)	0.095/**0.096** (**0.091**/0.075)	1.32/**1.34** (1.28/**1.32**)
	Medium	132	0.36/**0.37** (-0.03/**0.21**)	0.10/0.10 (**-0.01**/0.05)	**0.134**/0.128 (0.105/**0.107**)	1.31/**1.32** (1.25/**1.26**)
	Acceptable	239	**0.61**/0.34 (0.15/**0.19**)	**0.33**/0.22 (**0.17**/0.14)	**0.117**/0.098 (**0.093**/0.076)	1.36/**1.37** (1.30/**1.31**)
	Incorrect	261	**0.21**/0.18 (-0.19/**0.03**)	**0.39**/0.21 (**0.23**/0.10)	**0.071**/0.054 (**0.053**/0.036)	1.35/**1.37** (1.30/**1.31**)
	Overall	643	**0.38**/0.28 (-0.03/**0.13**)	**0.30**/0.19 (**0.15**/0.10)	**0.102**/0.086 (**0.079**/0.066)	1.35/**1.36** (1.29/**1.30**)
**M-ZDOCK models for homo-complexes**	High	43	-0.02/**0.14** (-0.19/**0.06**)	-0.15/**-0.09** (-0.23/**-0.12**)	**-0.032**/-0.033 (-0.049/**-0.047**)	1.33/**1.36** (1.26/**1.30**)
	Medium	135	**0.81**/0.61 (**0.65**/0.50)	**0.20**/0.17 (**0.15**/0.13)	**0.058**/0.041 (**0.033**/0.022)	**1.38**/1.37 (**1.32**/1.29)
	Acceptable	138	**1.52**/0.90 (**1.12**/0.73)	**0.71**/0.52 (**0.54**/0.41)	**0.122**/0.070 (**0.094**/0.043)	1.34/**1.35** (1.28/1.28)
	Incorrect	136	**1.57**/0.85 (**1.22**/0.69)	**1.08**/0.63 (**0.86**/0.51)	**0.111**/0.050 (**0.090**/0.037)	1.28/**1.30** (1.21/**1.23**)
	Overall	452	**1.18**/0.73 (**0.88**/0.59)	**0.59**/0.39 (**0.44**/0.31)	**0.085**/0.046 (**0.061**/0.026)	1.33/**1.34** (1.27/1.27)

Table S2. Refinement results of GalaxyRefineComplex on the ZDOCK and M-ZDOCK models for the best of 10 refined models, for model 1 (in parentheses), for the mean of the 10 models (in pointy brackets).

**Test set**		**No. models**	**Mean improvement/Percentage of improved cases**			
			ΔL-RMSD	ΔI-RMSD	ΔF_{nat}	ΔMolP
**ZDOCK models for hetero-complexes**	High	18	0.31/78%<br>(-0.59/33%)<br><-0.23/50%>	0.04/78%<br>(-0.12/22%)<br><-0.05/39%>	0.069/94%<br>(0.045/89%)<br><0.044/89%>	1.31/100%<br>(1.24/100%)<br><1.22/100%>
	Medium	148	0.55/86%<br>(0.08/57%)<br><0.12/59%>	0.19/89%<br>(0.08/59%)<br><0.08/70%>	0.130/95%<br>(0.098/85%)<br><0.092/91%>	1.38/100%<br>(1.30/100%)<br><1.29/100%>
	Acceptable	248	0.92/87%<br>(0.23/59%)<br><0.23/63%>	0.45/86%<br>(0.24/65%)<br><0.17/68%>	0.125/96%<br>(0.096/88%)<br><0.084/92%>	1.43/100%<br>(1.35/100%)<br><1.29/100%>
	Incorrect	263	0.56/75%<br>(-0.07/49%)<br><0.02/49%>	0.42/81%<br>(0.11/57%)<br><0.10/60%>	0.070/84%<br>(0.050/71%)<br><0.044/80%>	1.42/100%<br>(1.34/100%)<br><1.33/100%>
	Overall	677	0.68/82%<br>(0.06/54%)<br><0.11/56%>	0.37/85%<br>(0.15/59%)<br><0.12/64%>	0.103/91%<br>(0.077/81%)<br><0.069/87%>	1.41/100%<br>(1.33/100%)<br><1.32/100%>
**M-ZDOCK models for homo-complexes**	High	45	0.50/89%<br>(0.18/69%)<br><0.23/78%>	0.03/62%<br>(-0.13/44%)<br><-0.06/53%>	-0.003/38%<br>(-0.016/33%)<br><-0.024/64%>	1.39/100%<br>(1.30/100%)<br><1.25/100%>
	Medium	134	1.09/90%<br>(0.77/78%)<br><0.66/77%>	0.29/86%<br>(0.17/77%)<br><0.16/74%>	0.072/73%<br>(0.045/63%)<br><0.041/62%>	1.40/100%<br>(1.31/100%)<br><1.30/100%>
	Acceptable	135	1.49/92%<br>(0.78/74%)<br><0.68/78%>	0.70/91%<br>(0.40/78%)<br><0.35/76%>	0.112/81%<br>(0.085/74%)<br><0.067/76%>	1.33/100%<br>(1.22/100%)<br><1.30/100%>
	Incorrect	131	1.60/85%<br>(1.00/68%)<br><0.68/71%>	1.12/89%<br>(0.73/71%)<br><0.56/75%>	0.102/78%<br>(0.077/65%)<br><0.051/66%>	1.33/100%<br>(1.21/100%)<br><1.22/100%>
	Overall	445	1.30/89%<br>(0.78/73%)<br><0.63/76%>	0.63/86%<br>(0.37/72%)<br><0.32/73%>	0.085/73%<br>(0.061/64%)<br><0.045/64%>	1.36/100%<br>(1.25/100%)<br><1.25/100%>

Table S3. Refinement results of GalaxyRefineComplex on the CAPRI hetero-complex targets for the best of 10 refined models and for model 1 (in parentheses).

Target	No. models (Initial model accuracy: ***/**/*/-)[1]	Mean improvement/Percentage of improved targets			
		ΔL-RMSD	ΔI-RMSD	ΔF_{nat}	ΔMolP
**Round22 T46**	6 (0/0/3/3)	0.32/83% (0.20/67%)	0.31/100% (0.17/100%)	0.092/100% (0.066/100%)	1.21/100% (1.05/100%)
**Round24 T50**	9 (0/3/3/3)	0.29/56% (0.08/44%)	0.11/78% (-0.02/44%)	0.194/89% (0.132/67%)	0.69/100% (0.64/100%)
**Round26 T53**	10 (1/3/3/3)	1.01/80% (0.56/80%)	0.61/90% (0.36/80%)	0.182/90% (0.133/90%)	0.68/100% (0.59/90%)
**Round26 T54**	6 (0/0/3/3)	0.44/83% (0.14/67%)	1.02/83% (-0.53/50%)	0.067/67% (0.056/50%)	0.98/100% (0.90/100%)
**Round30 T81**	3 (0/0/0/3)	0.36/67% (0.02/67%)	0.31/100% (0.12/67%)	0.017/67% (-0.006/60%)	1.16/100% (1.09/100%)
**Overall**	34 (1/6/12/15)	0.48/74% (0.20/65%)	0.47/90% (0.02/68%)	0.110/82% (0.076/61%)	0.95/100% (0.85/98%)

[1] The number of initial models with the accuracy level defined according to the CAPRI criterion, ***/**/* for models of high/medium/acceptable accuracy and – for incorrect models.

Table S4. Refinement results of GalaxyRefineComplex on the CASP11 homo-complex targets for the best of 10 refined models and the model 1 (in parentheses).

**Target**	**No. models (Initial model accuracy: ***/**/*/-)**[1]	**Mean improvement/Percentage of improved targets**			
		ΔL-RMSD	ΔI-RMSD	ΔF_{nat}	ΔMolP
**T69**	2<br>(0/1/1/0)	0.29/100%<br>(0.26/100%)	0.27/100%<br>(0.26/100%)	0.053/100%<br>(0.047/100%)	0.90/100%<br>(0.83/100%)
**T75**	3<br>(0/3/0/0)	0.47/100%<br>(0.34/100%)	0.23/100%<br>(0.19/100%)	0.012/67%<br>(-0.012/33%)	0.60/100%<br>(0.56/100%)
**T79**	1<br>(0/0/1/0)	-0.22/0%<br>(-0.37/0%)	0.18/100%<br>(0.03/100%)	-0.024/0%<br>(-0.083/0%)	1.07/100%<br>(1.07/100%)
**T80**	5<br>(0/3/2/0)	0.19/60%<br>(0.06/60%)	0.15/80%<br>(0.11/80%)	-0.006/60%<br>(-0.029/20%)	0.72/100%<br>(0.66/80%)
**T82**	4<br>(0/2/2/0)	-0.14/50%<br>(-0.18/50%)	-0.03/50%<br>(-0.04/50%)	0.036/50%<br>(0.031/50%)	1.47/100%<br>(1.38/100%)
**T84**	6<br>(0/3/3/0)	0.32/83%<br>(0.14/67%)	-0.04/67%<br>(-0.08/67%)	-0.039/17%<br>(-0.060/0%)	0.38/100%<br>(0.30/100%)
**T85**	6<br>(0/3/3/0)	-0.53/67%<br>(-1.01/67%)	-0.39/67%<br>(-0.49/67%)	-0.023/67%<br>(-0.026/67%)	0.29/67%<br>(0.24/50%)
**T87**	7<br>(0/3/3/1)	0.24/86%<br>(0.06/86%)	0.15/86%<br>(0.07/71%)	0.019/100%<br>(0.004/57%)	0.81/71%<br>(0.75/71%)
**T90**	6<br>(0/3/3/0)	-0.06/33%<br>(-0.16/17%)	0.03/50%<br>(0.00/33%)	0.049/83%<br>(0.039/67%)	0.54/100%<br>(0.52/83%)
**T91**	6<br>(0/3/3/0)	0.29/83%<br>(0.25/83%)	0.11/50%<br>(0.08/50%)	0.021/67%<br>(0.018/67%)	0.49/50%<br>(0.44/50%)
**T92**	6<br>(0/3/3/0)	0.07/50%<br>(0.03/50%)	0.08/83%<br>(0.05/83%)	0.024/100%<br>(0.021/83%)	0.69/67%<br>(0.65/67%)
**T93**	5<br>(0/3/1/1)	-0.10/60%<br>(-0.23/40%)	-0.03/60%<br>(-0.05/60%)	0.092/100%<br>(0.088/100%)	1.01/100%<br>(0.97/80%)
**T94**	3<br>(0/0/3/0)	0.25/67%<br>(0.17/67%)	0.21/67%<br>(0.12/67%)	0.128/100%<br>(0.078/100%)	1.16/100%<br>(1.10/100%)
**Overall**	60<br>(0/30/28/2)	0.08/65%<br>(-0.05/60%)	0.07/74%<br>(0.02/71%)	0.026/70%<br>(0.008/57%)	0.78/89%<br>(0.73/83%)

[1] The number of initial models with the accuracy level defined according to the CAPRI criterion, ***/**/* for models of high/medium/acceptable accuracy and – for incorrect models.

Table S5. Blind prediction results by GalaxyRefineComplex on the targets of CAPRI round 30. Model quality for the best of the 10 submitted models and that for the initial model are compared. Improvements are shown in parentheses.

**Target**[1]	**Accuracy before**[4]**/after refinement**			
	**L-RMSD**	**I-RMSD**	**F_{nat}**	**MolP**
**T75**[2]	6.76/7.37	2.62/2.65	0.301/0.452	2.74/2.34
	(-0.61)	(-0.02)	(0.151)	(0.40)
**T79**[2]	5.90/5.32	3.46/3.20	0.346/0.346	2.28/2.16
	(0.58)	(0.26)	(0.000)	(0.12)
**T80**[2]	2.53/2.46	1.78/1.73	0.568/0.623	3.66/2.58
	(0.07)	(0.05)	(0.055)	(1.08)
**T84**[2]	2.78/2.75	2.06/1.96	0.672/0.748	3.67/2.65
	(0.03)	(0.10)	(0.076)	(1.02)
**T85**[2]	3.78/1.94	1.85/1.63	0.663/0.731	3.97/2.70
	(1.84)	(0.22)	(0.068)	(1.27)
**T87**[2]	2.65/2.58	2.48/2.42	0.408/0.443	3.64/2.68
	(0.07)	(0.06)	(0.035)	(0.97)
**T90**[2]	2.83/2.95	3.32/3.31	0.376/0.420	3.67/2.76
	(-0.11)	(0.01)	(0.044)	(0.91)
**T91**[2]	5.95/5.91	3.91/3.91	0.521/0.604	3.88/2.68
	(0.04)	(0.00)	(0.083)	(1.20)
**T92**[2]	7.75/7.87	5.59/5.38	0.358/0.415	3.86/2.84
	(-0.12)	(0.21)	(0.057)	(1.02)
**T93**[2]	5.14/5.08	2.56/2.46	0.418/0.612	3.71/2.64
	(0.06)	(0.10)	(0.194)	(1.07)
**T94**[2]	9.15/8.78	3.86/3.65	0.389/0.500	3.73/2.97
	(0.37)	(0.21)	(0.111)	(0.76)
**T81**[3]	5.61/5.61	1.72/1.80	0.811/0.784	2.65/1.83
	(0.00)	(-0.08)	(-0.027)	(0.82)
**T89**[3]	4.15/4.87	2.10/2.09	0.491/0.623	2.07/1.85
	(-0.72)	(0.01)	(0.132)	(0.22)
**Overall**	5.00/4.88	2.87/2.78	0.486/0.562	3.35/2.51
	(0.12)	(0.09)	(0.075)	(0.83)
**Improvement**	62%	77%	85%	100%

[1] Targets for which "Seok" group performed refinement predictions during the CAPRI round 30.

[2] Homo-complex targets

[3] Hetero-complex targets

[4] Initial models for homo-complexes were generated by GalaxyGemini (Lee, *et al.*, 2013).

Table S6. Refinement results with and without consideration of interface residues are presented for the best of 10 models. The best results are shown in bold and percentage of improved targets is shown in parentheses.

**Training Set**		**No. models**	**Mean improvements with/without interface consideration**		
			ΔL-RMSD	ΔI-RMSD	ΔF_{nat}
**ZDOCK models for hetero-complexes**	High	11	**0.275** / 0.096 (73% / **75**%)	**0.013** / -0.008 (55% / **80**%)	**0.100** / 0.050 (**91**% / 88%)
	Medium	132	**0.619** / 0.170 (**86**% / 82%)	**0.149** / 0.028 (**82**% / 55%)	**0.141** / 0.095 (**97**% / 64%)
	Acceptable	239	**0.831** / 0.194 (**84**% / 76%)	**0.383** / 0.102 (**86**% / 77%)	**0.124** / 0.083 (**96**% / 90%)
	Incorrect	261	**0.485** / 0.204 (72% / **73**%)	**0.448** / 0.132 (**83**% / 79%)	**0.075** / 0.047 (88% / **94**%)
	Overall	643	**0.637** / 0.192 (**79**% / 77%)	**0.335** / 0.097 (**84**% / 82%)	**0.107** / 0.071 (**93**% / 82%)

FIGURES

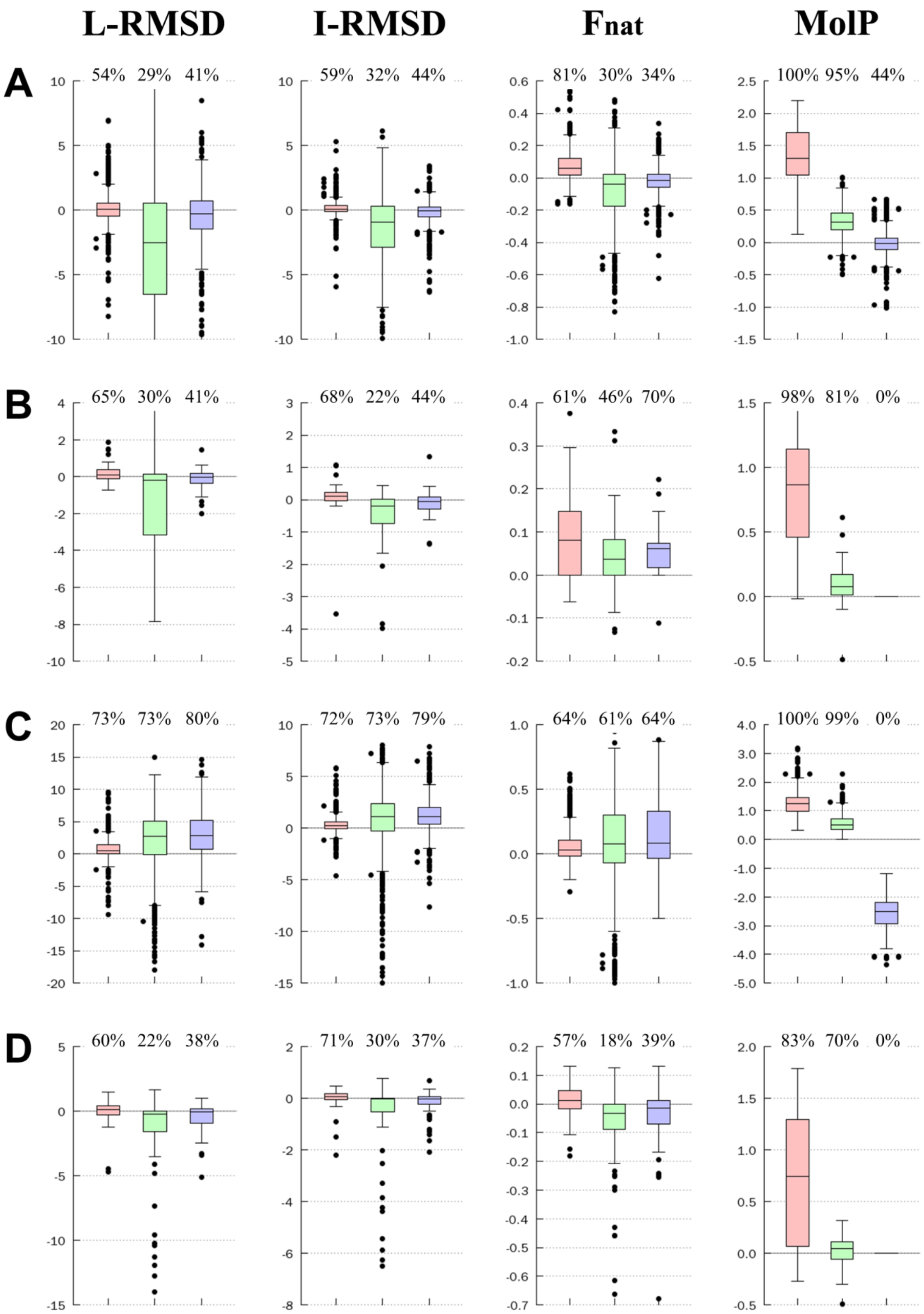
L-RMSD
I-RMSD
Fnat
MolP
A
54% 29% 41%
59% 32% 44%
81% 30% 34%
100% 95% 44%
B
65% 30% 41%
68% 22% 44%
61% 46% 70%
98% 81% 0%
C
73% 73% 80%
72% 73% 79%
64% 61% 64%
100% 99% 0%
D
60% 22% 38%
71% 30% 37%
57% 18% 39%
83% 70% 0%

Figure S1. Qualities of the model 1s generated by GalaxyRefineComplex (red), RosettaDock (green), and FiberDock/SymmRef (blue) are presented in boxplots. Results for (A) ZDOCK models for the hetero-complexes of the ZDOCK Benchmark 4.0, (B) CAPRI models for hetero-complexes, (C) M-ZDOCK models for the homo-complexes of the PISA benchmark set, and (D) CAPRI models for homo-complexes.

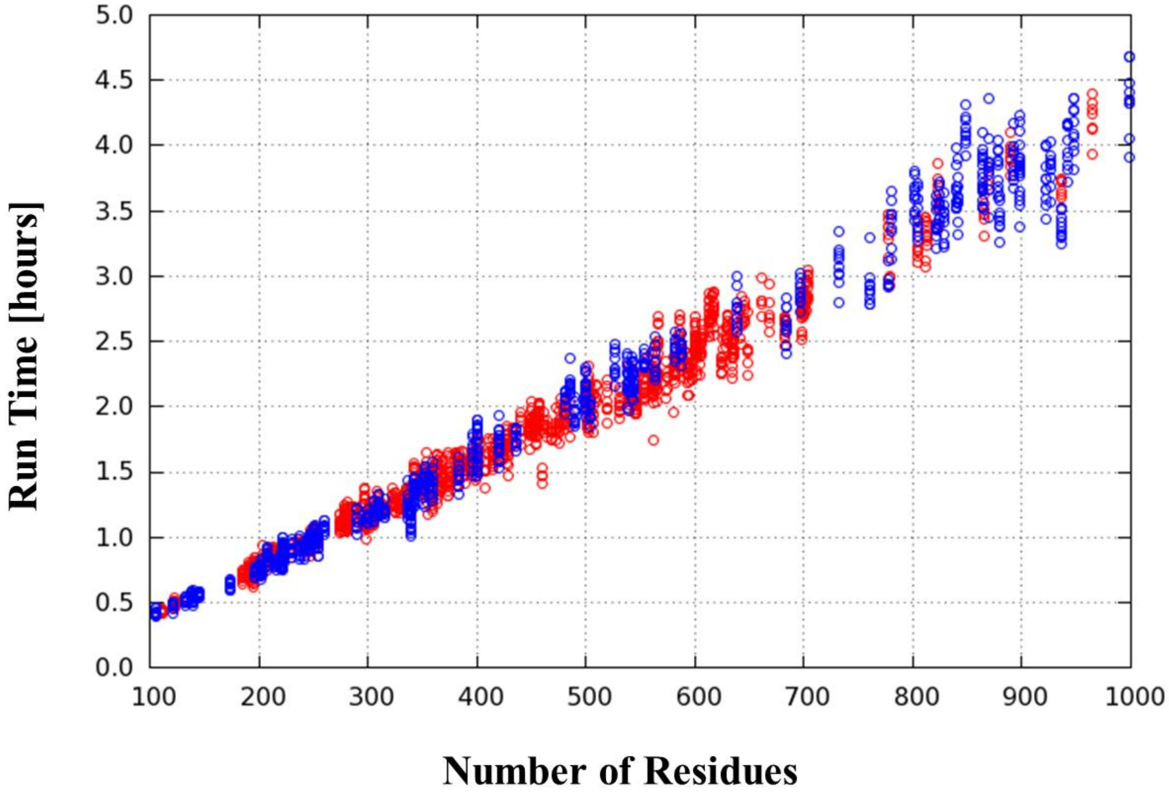

Figure S2. GalaxyRefineComplex run time as a function of the number of residues. Run times for hetero- and homo-complex structures are shown in red and blue, respectively. The computer time is measured on an in-house Linux cluster composed of 16 cores of Intel Xeon E5410 (2.33 GHz) processors.
